# Utilisation of Exhausted Coffee Husk as Low-Cost Bio-Sorbent for Adsorption of Pb^2+^

**DOI:** 10.21315/tlsr2022.33.3.12

**Published:** 2022-09-30

**Authors:** Rusnam, Aninda Tifani Puari, Nika Rahma Yanti, Efrizal

**Affiliations:** 1 Department of Agricultural and Biosystem Engineering, Faculty of Agricultural Technology, Andalas University, Padang, West Sumatera, Indonesia; 2 Department of Biology, Faculty of Mathematics and Natural Science, Andalas University, Padang, West Sumatera, Indonesia

**Keywords:** Adsorption, Biochar, Chemical Activation, Exhausted Coffee Husk, Ion Pb^2+^

## Abstract

This study utilised a bio-sorbent from exhausted coffee husk (ECHBS) for the removal of ion Pb^2+^ from an aqueous solution. Four different activation methods were conducted by chemical activation with KOH, H_3_PO_4_, ZnCl_2_, and without chemical activation. In addition, the influence of process parameters such as heating temperature, heating time and heating gradient were investigated. Based on the experimental results, ECHBS without chemical activation (biochar) had the highest Pb^2+^ ion removal efficiency. The results showed that the heating temperature of 500°C, the heating time of 60 min and the heating rate of 15°C/min were optimum for preparation of the biochar. Under the optimum conditions, the removal efficiency and adsorption capacity reached 99% and 3.3 mg/g, respectively. The experimental data indicated that the adsorption isotherms are well fitted with the Langmuir Equilibrium isotherm model. Furthermore, the adsorption of the biochar follows the pseudo-second-order model. The result obtained from the present study confirmed that exhausted coffee husk is a suitable low-cost bio-sorbent for removing ion Pb^2+^.

**Highlights**
- The activation methods of exhausted coffee husk (ECH) were differentiated based on activation agent used, ZnCl_2_, KOH, H_3_PO_4_ and no chemical activation.
- No chemical activation method was the most suitable activation method of ECH as biosorbent for Pb^2+^ removal in solution.
- The optimum carbonisation parameters for ECH biochar were identified to obtain the effective removal of Pb^2+^ ion.

## INTRODUCTION

Pollution of the aquatic environment by heavy metal ions is currently one of the major environmental issues due to its toxicological problem to the environment and living organisms (Abbas *et al*. 2016; Oliveira *et al*. 2008). The previous study has listed the toxicity of certain material ions, and Lead [Pb (II)] is considered as one of the heavy metal ions (Akpor 2014). Apart from the main natural sources, Pb (II) pollution in wastewater streams is also caused by human activities, such as metal finishing and electroplating, mining, textile activities, paint and car radiator manufacturing, as well as from agricultural practices (Akpor 2014; Smith *et al*. 1996).

Pb (II) contaminated wastewater may be toxic for humans when the ion gets accumulated in the soft tissue of the body. Pb (II) is considered the number one health threat to humans, particularly to children, and its effects can last a long time (Duruibe & Egwurugwu 2007; Salem *et al*. 2000). Moreover, the metal ion is non-degradable and persistent in the environment (Abbas *et al*. 2016). Thus, removing Pb (II) from wastewater is essential to protect the environment and public health. Some reliable and economically feasible technologies have been significantly developed to remove Pb (II) ions from an aquatic environment.

One of the alternative processes that has gained attention in metal pollution control is biosorption. The production of bio-sorbents (BS) in the form of biochar (BC) or activated carbon (AC) has been extensively studied in the last decade due to its potential application for the removal of heavy metals from wastewater (Gadd 2009; González-García 2018; Pollard *et al*. 1992; Ukanwa *et al*. 2019; Volesky & Holan 1995; Zhou *et al*. 2019). González-García (2018) summarised that several criteria should be considered when selecting a potential precursor for the development of BS. Regarding the criteria, abundant natural material would satisfy all the requirements; however, following Pollard *et al*. (1992), the selection based on raw material availability is often preferable. Furthermore, Evans *et al*. (1999) pointed out that the manufacture of AC should balance economic viability with a minimum of resources used in the process of conversion. Therefore, based on these criteria, agricultural residues from coffee industries are a potential candidate for BS (Djati Utomo & Hunter 2006; Pandey *et al*. 2000).

**Figure 1:**
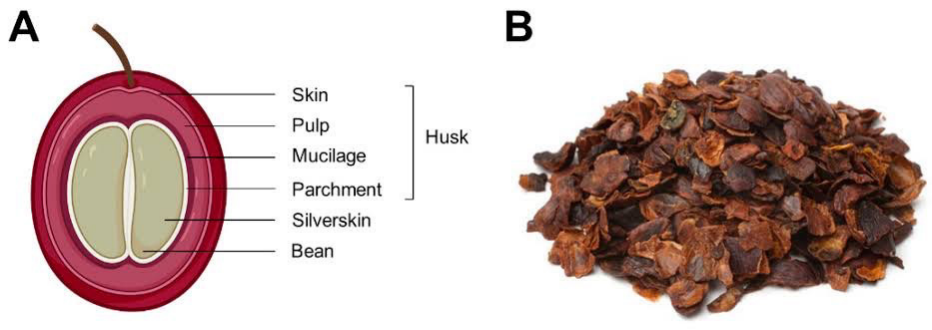
Coffee been parts (Rebollo-Hernanz *et al*. 2021)

Coffee industries generate significant amounts of agricultural waste in the form of coffee by-products. Coffee husks (CH), comprised of the dry outer skin, pulp, and parchment (Fig. 1), are probably the major residues from the handling and processing of coffee (Oliveira *et al*. 2008). Reportedly, processing 700 thousand tons of coffee beans could generate 420 thousand tons of agricultural solid waste. Furthermore, approximately one ton of CH is generated for every ton of coffee bean produced. In Indonesia, particularly, as the fourth largest coffee producer, the production of coffee in the last four years ranged from 600,000 to 761,000 metric tons (International Coffee Organization 2020; TheCoffeeGuide.Org, n.d.). Hence, processing CH into a valuable product has proposed a solution to the agricultural solid waste problem.

To this date, CH has been generally processed into fertiliser (Torres-Valenzuela *et al*. 2019), fuel (Rathinavelu & Graziosi 2005), and BS (Ayalew & Aragaw 2020; Berhe *et al*. 2015). Recently, CH has also been processed into an infusion drink called Cascara tea (Agavi 2020; Agronet.co.id, 2020). Likewise, its potential food production adds value to CH as a coffee by-product. However, disposal of the cascara tea also constitutes a major solid waste problem in a form of exhausted coffee husk (ECH). Furthermore, to the best of our knowledge, CH residue has no profitable uses (Bondesson 2015; Klingel *et al*. 2020). Hence, for moving towards circular economy, utilising BS from ECH for giving additional value and reusing the residue of CH should be prioritised.

Many scholars have done a lot of studies on BS under various pyrolysis and activation conditions. The results showed that activation parameters (Rufford *et al*. 2010; Ukanwa *et al*. 2019), heating temperature (Ayalew & Aragaw 2020; Lee *et al*. 2014), heating time (Örkün *et al*. 2012), and heating rate (Zhou *et al*. 2019) are important factors in the preparation of BS. For instance, the yield, surface area, pore-volume and functional group are varied with the different parameters.

This present study aims to observe the effect of the respective factors on the properties of BS from ECH for removal of Pb (II) from aqueous solution through batch mode adsorption technique. In addition, the adsorption isotherm and adsorption of the ECH have also been studied.

## MATERIALS AND METHODS

### Raw Material and Chemicals

Dry ECH was collected from the local cascara processing unit, PT. AGAVI, West Java, Indonesia. The ECH was obtained after the infusion process before the drying process under the sun. Meanwhile, Pb (II) working solutions (20 mg/L–100 mg/L) were prepared by diluting the stock solution Pb(NO_3_)_2_ (1,000 mg/L) with ultrapure water prior to the batch adsorption experiment. All chemical agents were of analytical grade. Pb(NO_3_)_2_ stock solutions, H_3_PO_4_, KOH and ZnCl_2_ were purchased from Merck, KGaA, Darmstadt, Germany.

### Bio-sorbent Preparation

First, dry ECH was washed with distilled water to remove dirt and colour, then dried in a drying oven (MEMMERT UN 55) at 105°C for 4 h. For BS in the form of AC, the dried ECH were activated prior to carbonisation with three activating agents representing the acid (H_3_PO_4_), base (KOH) and salt (ZnCl_2_). Dried ECH was impregnated with KOH, H_3_PO_4_ (85%), and ZnCl_2_ with the ratio of 1:1 for 24 h using an incubated shaker (INCU-Shaker 10L) under the temperature of 60°C and shaken at 100 rpm. After the reaction subsided, the impregnated ECH were dried in a drying oven at 105°C for 24 h. The activation process of ECH combined the methods by Ayalew and Aragaw (2020) and Zhou *et al*. (2019). The dried activated ECH was carbonised at 400°C with Muffle Furnace (Nabertherm B180) for a heating time of 1 h and a heating rate of 5°C per min. A ceramic crucible bowl was used for the muffle furnace. During the process, the ceramic crucible bowl was wrapped with aluminum foil to isolate the oxygen. The activated powder was neutralised with excess hot distilled water until the pH of 6–8 was achieved. Then it was dried at 105°C for 2 h and kept in a plastic bag placed in a desiccator for the subsequent experiment and adsorbent characteristic.

### Single-Factor Experiments

A series of single-factor experiments were conducted to study the effect of three factors on the adsorptive property of ECH. For this purpose, the removal efficiency (RE) and adsorption capacity (*q_t_*) of Pb^2+^ on the activating agent, heating temperature, heating time, and heating rate were investigated. Various heating temperatures (300°C–600°C), heating time (15 min–120 min), and heating rate (5°C/min–15°C/min) were applied during the production process, respectively.

### Preparation of Pb (II) Solution

**Figure 2:**
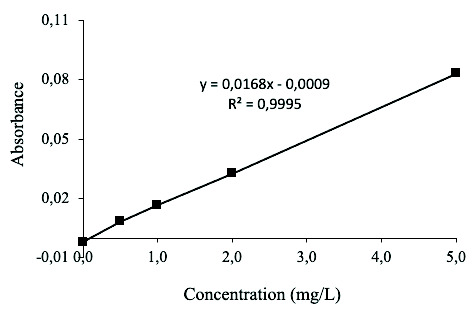
Calibration curve of Pb (II) solution at different concentration

Pb (II) standard solutions (0, 0.5, 1, 2, 5 mg/L) were prepared by diluting the Pb(NO_3_)_2_ stock solution with ultrapure water. Their concentrations were measured by Shimadzu AA-7000 atomic absorption spectrophotometer (AAS) (Shimadzu, Japan). A linear calibration curve was obtained from absorbance values versus the respective concentrations of Pb (II) solutions, as shown in Fig. 2.

### Biosorption Studies

The adsorption performance of all prepared ECH was tested in batch adsorption experiments. The batch experiments of Pb^2+^ were performed in 250 mL Erlenmeyer flask containing 100 mL of Pb^2+^ ion solutions. The solutions were shaken at a speed of 50 rpm/min and a contact time of 40 min at 295 K. The dosages of BS were varied (0.2, 0.4, 0.6, 0.8, 1 g), with initial concentration of Pb^2+^ in all single factor experiments solutions were 20 mg/L. At the end of each experiment, the supernatant was drawn out and subsequently filtered with filter paper. Following that, the solution was diluted with varied dilution factors, and the concentration of Pb^2+^ was measured with AAS. The detected concentration was multiplied with the dilution factor to determine the amount of Pb^2+^. The Pb^2+^ adsorbed was determined by the difference between the initial concentration and the concentration of the filtrated solution after each experiment.

The RE (%) of Pb^2+^ and the *q_t_* (mg/g) were determined using Equations (1) and (2), respectively (Zhou *et al*. 2019). The adsorption at the equilibrium of Pb^2+^ ion (*Q_e_*) (mg/g) was determined by Equation (3) (Ayalew & Aragaw 2020).


(1) $$Re=\frac{C_{o}-C_{t}}{C_{o}}\times 100$$

(2) $$q_{t}=\frac{\left(C_{o}-C_{t}\right)v}{m}$$

(3) $$Q_{e}=\frac{\left(C_{o}-C_{e}\right)v}{m}$$

where *C_o_* is the concentration of Pb^2+^ at initial, *C_t_* is the concentration of Pb^2+^, *C_e_* is the concentration of Pb^2+^ at equilibrium, *V* is the solution volume of Pb^2+^ (mL) and *m* is the adsorbent dosage (g).

### Adsorption Models

#### Isotherm models

The Langmuir parameter was determined by plotting the Langmuir Equation (4) into the linear form as given by Equation (5).


(4) $$q_{e}=\frac{q_{m}kl\;Ce}{1+Kl\;Ce}$$

(5) $$\frac{1}{Q_{e}}=\frac{1}{Q_{m}}+\frac{1}{Kl\;Qm\;Ce}$$

Ayalew and Aragaw (2020) identified the values of *Q_m_* and *Kl* were computed from the Langmuir plot, where the intercept for 1/*C_e_* versus the slope for *Q_e_/C_e_*. The correlated coefficient (R^2^) value was obtained from both model parameters. *Q_e_* is the concentration of ion Pb^2+^ at equilibrium in solution (mg/L) while *Q_m_* is the ion concentration when monolayer forms on the BS (mg/g). Separation factor (*R_L_*) was also expressed from the isotherm model, which was obtained from dimensionless equilibrium factor mentioned in Equation 6.


(6) $$R_{L}=\frac{1}{1+KL\;C_{o}}$$

where *KL* is the Langmuir constant (L/mg) related to the affinity of binding sites and the free energy of sorption and *C_o_* is the highest initial Pb^2+^ ion concentration (mg/L). The calculated *R_L_* was later interpreted into the value of R_L_ and its favourability adsorption in as shown in Table 1 (Ayalew & Aragaw 2020).

The applicability of Freundlich isotherm adsorption was analysed with the same set of experimental data, where the parameters were determined by expressing the Freundlich Equation (7) into linear form, as mentioned in the Equation (8):


(7) $$Qe=Kf\;\chi \sqrt[{n}]{C}$$

(8) $$1n\;qe=1n\;kf+\left(\frac{1}{n}\right)*lnCe$$

*Kf* and *n* are the Freundlich adsorption isotherm constants, where *kf* indicates the adsorption capacity and *n* determines the intensity of the biosorption. The Freundlich constants were obtained from the slope and intercept of a plot of log (*qe*) versus log (*Ce*) (Ayalew & Aragaw 2020; Kim & Kim 2020).

### Adsorption kinetics

Pseudo-first-order and pseudo-second-order kinetics models were used to analyse the adsorption kinetics of Pb^2+^ on the ECHBS. Predicted values were represented in correlation coefficient for comparing the experimental data and model. The more relative the value obtained from two orders plotted, the more applicable is the kinetic model of Pb^2+^ on the ECHBS.

Equation 9 expressed the pseudo-first-order and applying the boundary condition *t* = 0 to *t* = *t* and *qt* = 0 to *qt* = *qt*, it gave the Equation (10):


(9) $$\frac{dqt}{dt}=kl\left(qe-qt\right)$$

(10) $$\text{loq}(qe-qt)=\text{loq }qe-\left(\frac{kl}{2.303}\right)t$$

Where *qe* (mg/g) is the amount of Pb^2+^ ion adsorbed at equilibrium, *qt* (mg/g) is the amount of Pb^2+^ ion adsorbed at particular time *t* (min) and *kl* (min^−1^) is the rate constant of the pseudo-first-order adsorption. To obtain the rate constant, the value of log (*qe* − *qt*) was linearly plotted with time (*t*). The value of *Kl* and predicted *qe* were obtained from the slope and intercept of the plot. Meanwhile, the pseudo-second-order model was expressed in Equation (11) , and later, the linearised form of the pseudo-second-order was extracted in terms of Equation (12).


(11) $$\frac{dqt}{dt}={kl}^{2}{\left(qe-qt\right)}^{2}$$

(12) $$\frac{t}{qt}=\frac{1}{k_{2}*qe^{2}}+\left(\frac{1}{qe}\right)t$$

Likewise, the value of K_2_ g/mg min) was extracted from the slope while the value of *qe* from the intercept of the graph *t/qt* versus *t* (Bhattacharyya & Gupta 2008).

### Characterisations and Analysis Methods

The Pb^2+^ concentration was measured using Shimadzu AA-7000 AAS (Shimadzu, Japan), and the pH of the solution was determined by Thermo Scientific Orion Star A211 pH meter (Thermo Fisher, USA). The surface morphology and element composition of BS were imaged using Hitachi S-3400N scanning electron microscopy (SEM) with energy dispersive X-ray (EDX) spectroscopy (Horiba, Japan). The functional groups on BS surfaces were recorded by the IRTracer-100 Fourier Transform Infrared Spectrophotometer (FT-IR) (Shimadzu, Japan). The composition and structure of the atoms or molecules inside OECSBC BS of ECH were characterised by XRD with PANalytical X’Pert PRO series X-ray (powder) diffractometer (PANalytical, the Netherlands). The specific surfaces area and pore size of the BS were analysed by Brunauer-Emmett-Teller (BET) surface analyser (TriStar II 3020 2.00). The data and figures of experiments were analysed by OriginPro 2019.

## RESULTS AND DISCUSSIONS

### BS Preparation

To explore the effect of chemical activator on property of BS from ECH (ECHBS), four experiments with different chemical activation methods. The methods were carried out using KOH (base), H_3_PO_4_ (acid), ZnCl_2_ (salt) and without chemical activator. The experiments were done by impregnating the ECH with the respective chemical activation methods by the ratio dried ECH to the chemicals of 1:1 at 60°C for 24 h prior to pyrolysis process. The pyrolysis processes were done at temperature of 400°C and heating rate of 5°C/min.

#### Effect of activating agent

**Figure 3:**
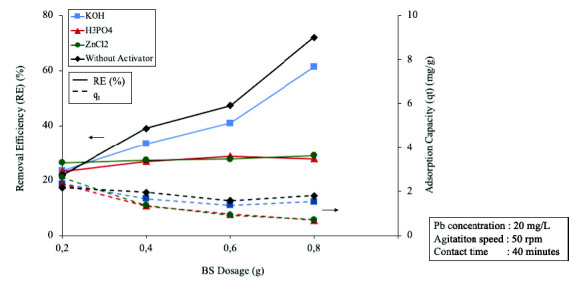
Removal efficiency (RE) and adsorption capacity (*q_t_*) of the BS from the ECH with different activating agents at different BS dosages.

The result of ECHBS with varied chemical activation methods were evaluated by the amount of Pb (II) ion removed during the biosorption studies. Fig. 3 shows the removal efficiency (RE) and adsorption capacity (*q_t_*) of the ECHBS at the varied BS dosages for all the methods.

It can be seen that the RE of ECHBS generally increased with the increasing of BS dosage. According to Babel and Kurniawan (2004), the result can be explained by the fact that with the increasing amount of BS dosage in the solution, greater number of exchangeable sites for metal ions are available. A significant difference with regards RE of ion Pb was also observed in Fig. 3 between ECHBS without chemical activation and the chemically modified ones. In Fig. 3, the RE gradually increased with the increase of ECHBS with no chemical activation and KOH, while that for H_3_PO_4_ and ZnCl_2_ slightly increased. Of the three types of chemically activated ECHBS, chemically modified with KOH has the highest RE compared to other two with 61.2% at the highest dosage of ECHBS. Whereas, the highest RE achieved by ECHBS chemically activated with H_3_PO_4_ and ZnCl_2_ were only 27.8% and 29%, respectively. It was stated by González-García in 2018 that highly alkaline activating agents are preferable in terms of helping to develop the porosity by way of dehydration and degradation of the biomass structure. Hence, the higher RE by the ECHBS, chemically activated by KOH, was due to the more developed porosity. However, it is interesting to note that the greatest RE in the experiments was achieved by ECHBS without chemical activation, commonly called as biochar, with the highest RE 72% at the highest ECHBS dosage of 0.8 g.

Fig. 3 also demonstrated the adsorption capacity (*q_t_*) of ECHBS from the experiments for all activation methods. The *q_t_* showed a reverse trend to the RE for all the experiments. Generally, a decreased was observed in adsorption capacity by the increase of the dosage. The highest point of adsorption capacity was reached by ECHBS chemically activated by ZnCl_2_ with 2.6 mg/g at the dosage of 0.2 g. However, in general the highest *q_t_* was achieved by ECHBS with no chemical activation. The capacity of biochar reduced from 2.2 to 1.7 mg/g when the mass of ECHBS added to the solution was increased from 0.2 to 0.8 g. Mohd Salim *et al*. (2016) states that the decrease in the q_t_ is explained by the saturation of bio-sorbent by bio-sorbate. In general, the available amount of biosorption sites is limited to the mass of BS. Hence, the greater the amount of BS mass, the higher quantity of biosorption sites. Concerning RE and adsorption capacity results, therefore, no chemical activation was selected as the biosorption activation method for utilising ECH as BS.

### Surface Characterisation

#### Surface porosity morphology biosorption

**Figure 4:**
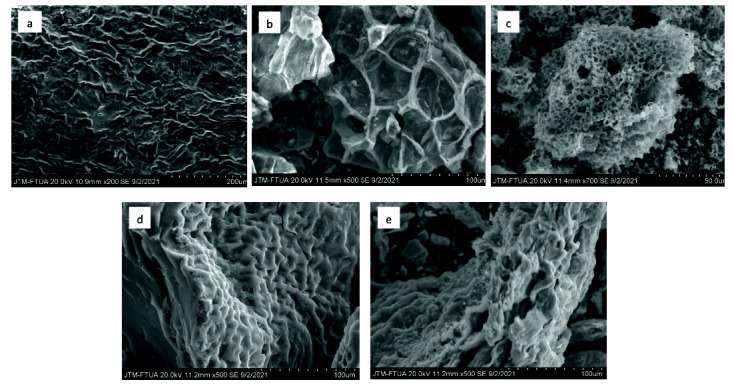
SEM images of (a) raw dried-ECH, (b) biochar and chemically activated carbons with (c) KOH, (d) H_3_PO_4_, (e) ZnCl_2_.

The scanning electron microscope (SEM) images of the surface morphology of the produced ECHBS are illustrated in Fig. 4. All the activation methods used in the experiments lead to transformation of a non-porous surface area precursor into a porous material with visible pores of varying size. The open-pore structure provides maximum access for the ion into the surface area of the activated carbon through enhanced diffusion. The varying size of pores resulted in different diffusion rates (Djilani *et al*. 2015).

The results showed that the activation process chosen had a significant impact on ECHBS porosity, particularly with the addition of chemical activation agents. SEM images of biochar (Fig. 4 b) showed that the pores formed through thermal activation still retained the cell wall structures of the parent materials. However, the biomass derived chemically activated carbon (Figs. 4c, 4d and 4e) did not show any distinctive pore structure. Oh *et al*. (2003) explained that the lignin helps to sustain the morphology of biomass as it consists of many ester and ether bonds in its crosslinked structure. Therefore, the destruction of lignin components of the precursor during the impregnation process can be attributed to the non-distinctive porous structures shown by the biomass derived chemically activated carbons. A similar finding was also reported by Oginni *et al*. (2019) for the BS obtained from biomass derived chemically activated carbon.

#### FTIR analysis

**Figure 5:**
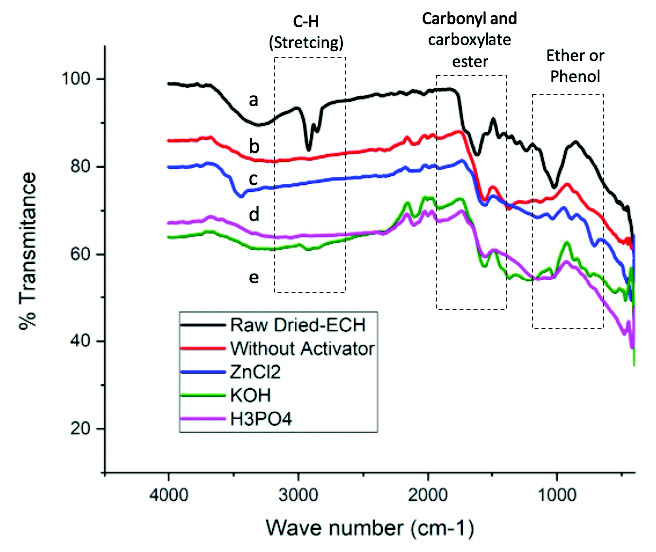
FTIR spectra of (a) biomass precursor (raw dried-ECH), (b) biochar (without activator), biomass derived chemically activated carbon with (c) ZnCl_2_, (d) H_3_PO_4_ and (e) KOH.

The knowledge of surface functional groups would give insight into the adsorption capability of the produced activated carbon. Therefore, fourier transform infrared spectra (FTIR) were collected for qualitative characterisation of surface functional groups of biomass precursor, biochar and biomass derived chemically activated carbon. The FTIR spectra obtained with different activation methods are presented in Fig. 5.

Fig. 5 showed that spectra were slightly changed after the activation, both thermally or chemically. Nevertheless, the spectra showed similar peak at certain functional group band. It was clearly seen in the FTIR spectrum obtained from raw dried-ECH that small weak bands were appearing at about 2,921 and 2,850 cm^−1^. They were generally attributed to C-H (stretching) bands, however, these bands were not observed at the spectrum of biochar (Fig. 5b) and all chemically activated carbons (Figs. 5c–5e). Furthermore, the raw dried-ECH also presented predominant peak of phenolic ether band at about 1,016 cm^−1^ however, the peak was hardly found at the spectrums of biochar and all chemically activated carbons.

It can also be observed that all spectrums showed the peak intensities at 1,653 cm^−1^, which can be attributed to carboxylate ester showing deformation vibrations. The peak intensity was decreased considerably after a treatment with no chemical activation (Fig. 5 b), followed by ZnCl_2_ (Fig. 5 c), H_3_PO_4_ (Fig. 5 d), KOH (Fig. 5 e), respectively. The spectrum indicated that the lignin is damaged after the treatments, particularly by H_3_PO_4_ and KOH impregnation. Expectedly from the comparison, the lowest intensity of carboxylate ester was by the activated carbon with KOH (Fig. 5 e), while the highest was achieved by the biochar (Fig. 5 b). The spectrum could support the non-distinctive pores showed by surface morphology of the biochar and activated carbons, as the raw dried-ECH deformed considerably with the impregnation causing the extraction of lignin component.

### BET Surface Area and Pore Size Analysis

The textural properties of ECHBS with different activation methods were characterised with surface area and pore size analyser. Table 2 shows the BET surface area and average pore diameters of the activated carbons, biochar from ECH and the previous study. The BET surface area from this study ranged between 1.5 and 943.33 m^2^/g. The highest surface area was achieved by AC activated with H_3_PO_4_ with 943.33 m^2^/g. It was followed by the biochar and ECH with KOH with 7.87 and 2.21 m^2^/g, respectively. The activated carbon activated with ZnCl_2_ had the lowest BET surface area with 1.5 m^2^/g.

It can be observed from Table 2, activating ECH with H_3_PO_4_ resulted in significantly higher surface area compared to the other three activation conditions. Moreover, the surface area of the BS from CH with similar activating agents (H_3_PO_4_ and ZnCl_2_) in the previous studies resulted in higher surface area than in this study. It can be the result of different treatment of the ECH and CH, impregnation ratio and activation temperature applied. Notably, the pore size from this study were higher than in the previous studies (Table 2). In contrast with the surface area, the largest pore size from this study was achieved by the AC with ZnCl_2_, followed by ECH with no activation agent, and KOH with 52.08 nm, 41.19 nm and 27.29 nm, respectively. The activated carbon with H_3_PO_4_ had the smallest pore size with 3.33 nm. This could be the cause it resulted in poorer RE than the biochar although the surface area of H_3_PO_4_ was higher.

### Single Factor Experiments

#### Effect of heating temperature on biosorption property of ECHBS

**Figure 6:**
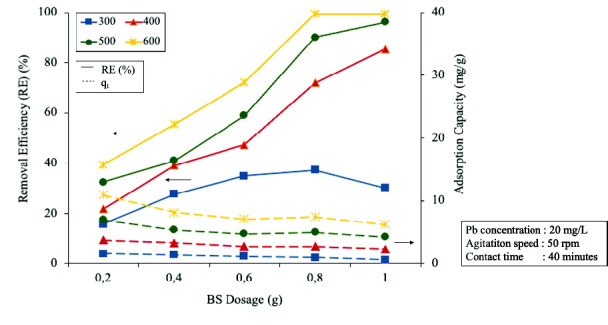
The effect of the different heating temperature on removal efficiency (RE) and adsorption capacity (*q_t_*) at all the BS dosages.

Several experiments were carried out to determine the effect of heating temperature on the property of the ECHBS biochar. The experiments were conducted under a range of temperatures from 300°C to 600°C, at a heating time of 1 h, heating rate 5°C/min and the varied BS dosages. The results are presented in Fig. 6.

**Figure 7:**
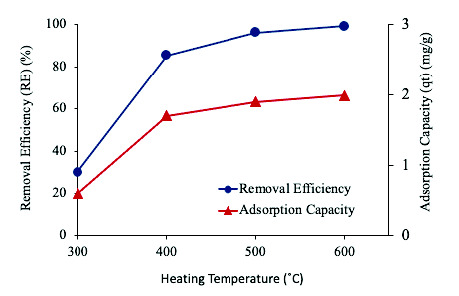
The effect of the different heating temperature on removal efficiency (RE) and adsorption capacity (*q_t_*) at the BS dosage of 1 g.

Fig. 6 shows that the RE and *q_t_* of Pb^2+^ by ECHBS increased with the increasing of heating temperature for all the dosages added. The highest RE of all the dosages was achieved at heating temperature of 600°C. However, it is interesting to note in Fig. 6 that at the highest dosage of 1 g, the RE between heating temperature of 500°C and 600°C did not increase. Therefore, to choose the optimum condition for the heating temperature applied, the RE obtained at ECHBS dosage of 1 g was plotted and compared. The results are shown in Fig. 7.

Fig. 7 presents the RE and *q_t_* of 1 g biochar on ion Pb^2+^ at different heating temperatures. It can be seen that when the heating temperature was increased from 300°C to 500°C, the RE and q_t_ were considerably increased. At the increased temperature, the RE increased from 30.1% to 96.1% while *q_t_* increased from 0.6 to 1.9 mg/g. Furthermore, when the temperature was raised to 600°C, the RE and q_t_ only slightly increased to 99.3% and 1.99 mg/g, respectively. Thus, although further increasing temperature to 600°C increased the adsorption effect compared to the effect of 500°C, the increase was only 3.2%. Considering the energy consumption when increasing the heating temperature, hence the 500°C was chosen as the optimum value of heating temperature for thermal activation of ECHBS biochar.

### Effect of Heating Time on Adsorption Property of ECHBS

**Figure 8:**
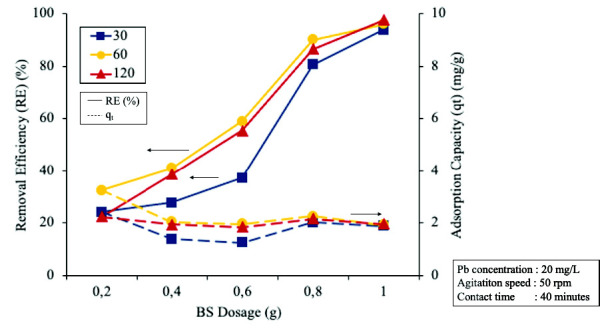
The effect of the different heating time on removal efficiency (RE) and adsorption capacity (*q_t_*) at all BS dosages.

To investigate the effect of heating time, some experiments were done at a range of times from 30 to 120 min at 500°C, heating rate of 5°C/min and the various BS dosages. The RE and *q_t_* obtained are presented in Fig. 8.

Fig. 8 shows that the RE and *q_t_* decreased by increasing the heating time. It was clearly seen that extending the heating time from 30 min to 60 min resulted in a considerable increase for all the dosages. The highest RE and *q_t_* in the experiments were achieved when the heating time was 60 min and the dosage was 1 g with 96% and 3.25 mg/g, respectively. However, when the heating time was extended from 60 to 120 min, the adsorption effect was slightly decreased for all the dosages. Extending the heating time longer than 60 min did not result in any higher adsorption effect. Thus, from the experimental data, 60 min was decided as the optimum heating time for the preparation of biochar from ECH.

### Effect of Heating Rate on Adsorption Property of ECHBS

**Figure 9:**
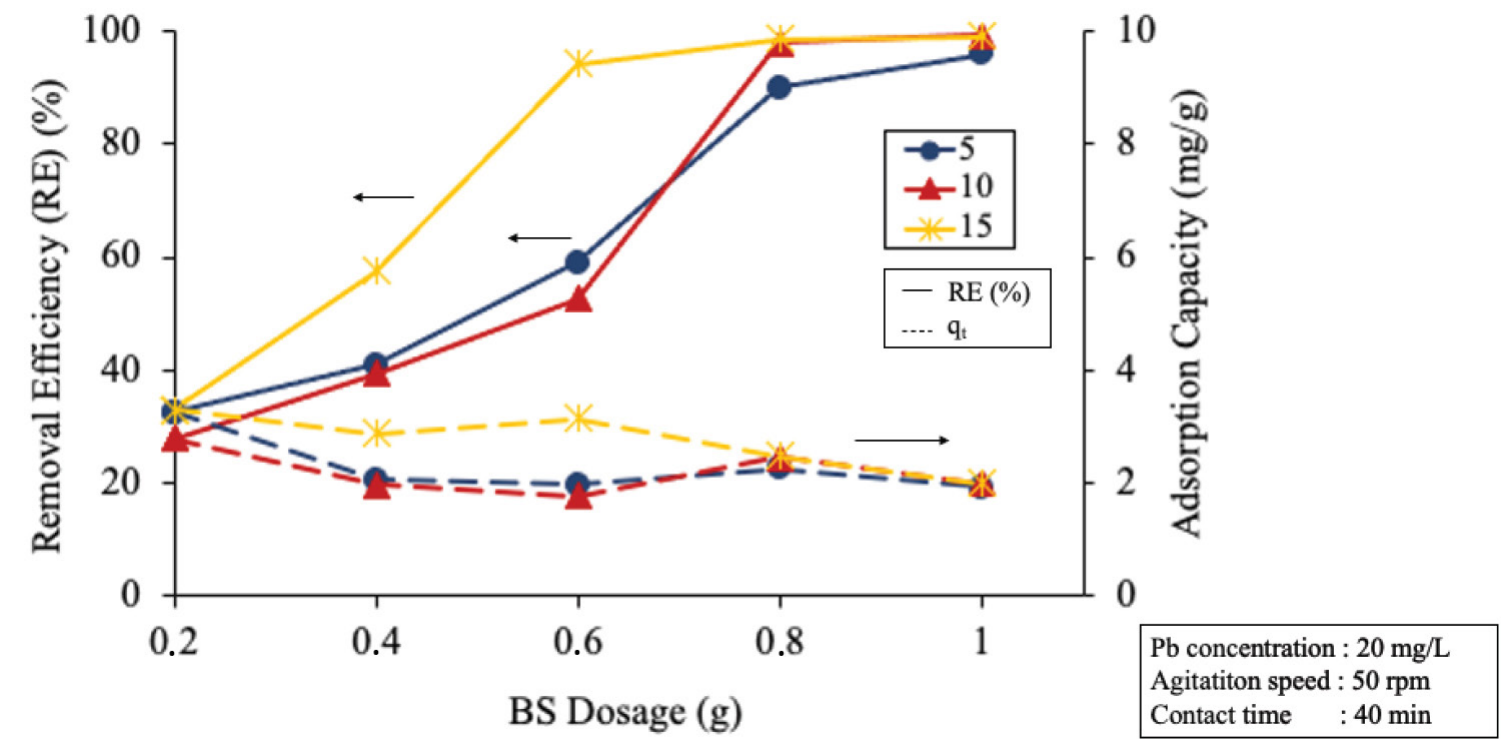
The effect of the different heating rate on removal efficiency (RE) and adsorption capacity (*q_t_*) at all the BS dosages.

The effect of heating rate on biochar property has been rarely studied. However, Zhou *et al*. (2019) reported in their study that heating rate has an effect on the property of the studied biochar. Hence, some experiments were conducted to investigate the effect of heating rate on the biochar from ECHBS. The experiments were done under different heating rates in the range of 5°C to 15°C/min at 500°C and varied BS dosages for 60 min of heating time. The results are presented in Fig. 9.

Fig. 9 shows the effect of heating rate on RE and *q_t_* obtained at various BS dosages. In general, the RE and *q_t_* increased by raising the heating rate. The highest RE and *q_t_* were achieved when the heating rate applied was 15°C/min for all the dosages. The differences of RE and *q_t_* for each heating rate are clearly seen in Fig. 9 when the dosage of 0.6 was added to the solution. When the heating rate was raised from 5°C to 10°C/min, the RE slightly decreased from 59% to 52%, while *q_t_* decreased from 1.96 to 1.76 mg/g. However, when the heating rate was increased from 10°C to 15°C/min, the RE increased to 94% while *q_t_* also increased to 3.14 mg/g. The highest RE of 99% and the highest *q_t_* of 3.30 mg/g were achieved when the heating rate of 15°C/min was applied.

### Adsorption Isotherm and Adsorption Kinetic of ECHBS

#### Adsorption isotherm

**Figure 10:**
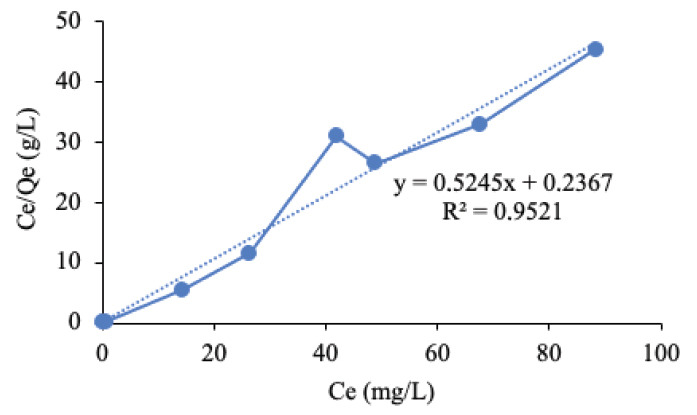

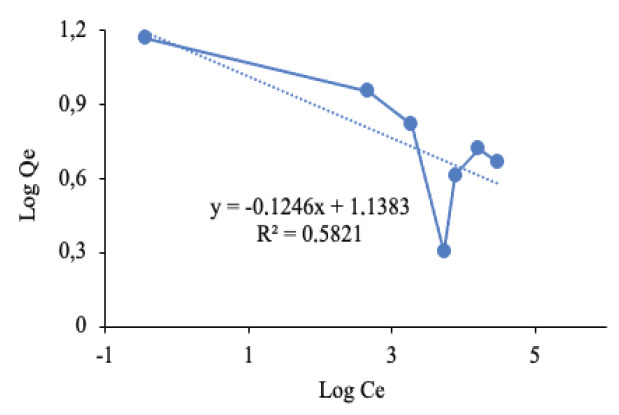
Adsorption isotherm for the removal of Pb^2+^ on the ECHBS (a) Langmuir and (b) Freundlich.

In this study, Langmuir and Freundlich model parameters were used to analyse the equilibrium data. The output from the model parameters provide important information on adsorption mechanism and surface properties of the BS (Ayalew & Aragaw 2020). Fig. 10 presents the model parameters of Langmuir and Freundlich along with the coefficient of determination (R^2^) of the linear plots.

The value of Q_m_ and K_L_ were extracted from the intercept and slope of the Langmuir plot (Fig. 10a), respectively. In addition, the value of R_L_ also calculated from the Equation (6) from the K_L_ obtained. Meanwhile, from the plotting of Freundlich isotherm (Fig. 10b) the magnitude exponent (n) was calculated to characterise the favourability of the model. The values obtained are presented in Table 3.

According to the values of R^2^ obtained in Table 3, the Langmuir isotherm was the best fit for the biochar from ECH. Furthermore, the value of R_L_ showed in Table 3 indicated it favourability adsorption referring to the type of separation of Langmuir isotherm in Table 1. Nevertheless, the Freundlich isotherm fitted did not show well for the biochar from ECH.

#### Adsorption kinetic

**Figure 11:**
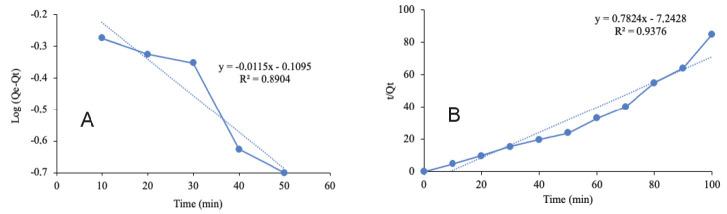
Adsorption kinetics of Pb^2+^ on the ECHBS: (a) pseudo-first-order and (b) pseudo-second-order rates.

The kinetic of ion Pb^2+^ biosorption by the biochar was expressed by plotting the experimental data. Fig. 11a presents the plotting for pseudo-first-order while Fig. 11b presents for pseudo-second-order with the linearised form for both orders.

The value of K_I_ and K_2_ were calculated from the slope of the graph for pseudo-first-order and pseudo-second-order, respectively. Meanwhile, the values of *q_e_* for both were extracted from the intercept of the graph. The calculated value from Equations (10) and (12) are mentioned in Table 4.

It can be seen in Table 4 that the correlated coefficient (R^2^) of the pseudo-second-order kinetic model (R^2^ = 0.9376) was greater than of the pseudo-first-order kinetic model (R^2^ = 0.8904). Hence, it can be concluded that the batch adsorption experiment of the biochar from ECH for the removal of ion Pb^2+^ follows the pseudo-second-order kinetic model. The same indication was also found in the previous studies of CH as BS for the removal of methylene blue (Ayalew & Aragaw, 2020) and ions of Ni^2+^ and Zn^2+^. Furthermore, the similar kinetic order was also found in for the removal of ion Pb^2+^ using mixed-waste activated carbon (ALOthman *et al*. 2016).

### Biosorption Mechanism of Pb^2+^ by ECHBS

For evaluating the RE of ion Pb^2+^ by the biochar from ECH, identification of the underlying mechanisms of the adsorption process is necessary. To study the adsorption mechanisms of the biochar on the ion, SEM-EDX, FTIR and XRD were used to analyse the biochar ECHBS before and after adsorption. It can be concluded that the adsorption mechanisms of ECHBS on Pb^2+^ mainly include surface physical adsorption and ion exchange.

### After and Before Adsorption SEM

**Figure 12:**
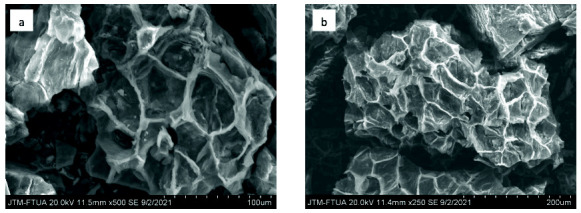
SEM images of ECHBS (a) before biosorption and (b) after biosorption.

SEM images provided only qualitative evaluation of the surface structure. The SEM images of ECHBS before and after biosorption are displayed in Fig. 12.

It can be seen from the Fig. 12 that SEM images of ECHBS before biosorption displayed a lot of stacked fragments. The stacked fragments allow the heavy metal ion, Pb^2+^, to enter and be trapped in the pores of the ECHBS. Fig.12b showed the SEM image of ECHBS after biosorption with smoother surface where the stacked pores were filled. According to the study by Zhou *et al*. (2019), the particle in the surface pores of the biochar after biosorption could be the heavy metal ion, which in our study was Pb^2+^ ion.

### EDX and XRD Results Indication

The EDX spectrums of ECHBS before and after biosorption of Pb^2+^ are presented in Table 5. It can be observed through elemental analysis with the EDX that the content of K, Ca and S decreased after the biosorption. Interestingly, Pb^2+^ ion was not detected in ECHBS after biosorption through elemental analysis with EDX. The reason for this could be that the highest adsorption capacity of ECHBS reached was 3 mg/g, which represented 0.3% Pb contained in the biochar.

**Figure 13:**
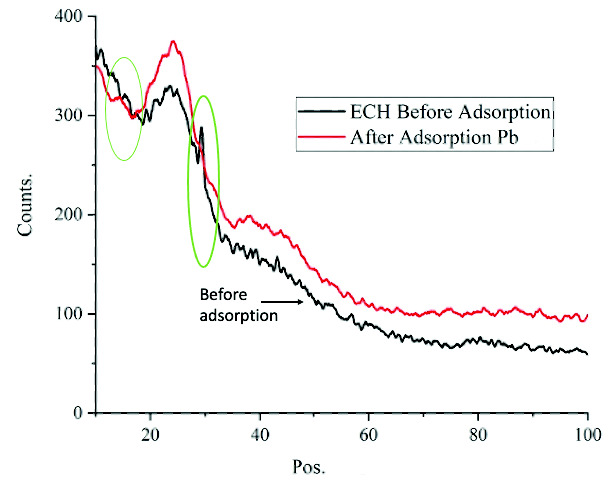
XRD patterns of ECHBS before and after adsorption of ion Pb^2+^.

Similar results were observed by Zhou *et al*. (2019) that the K and Ca ions play a role as an ion exchanger in biosorption. The XRD patterns of ECHBS before and after biosorption of ion Pb^2+^ also supported the findings. The XRD patterns of ECHBS before and after the biosorption are presented in Fig. 13. It can be seen in the Fig. 13 that there was a distinct sharp crystalline peak recorded at 2θ at 29°. A similar peak was also detected in XRD pattern of biochar in the study by Zhao and Nartey (2014). The study suggested that the peak recorded at 2θ at 29° indicated the presence of calcite (CaCO_3_). However, the crystalline peak was absent in the XRD pattern of ECHBS after the biosorption. These findings aligned with EDX data in Table 5, where Ca ion was absent in ECHBS after biosorption. In summary, Ca^2+^ and K^+^ ions had an ion exchange reaction with ion Pb^2+^.

Even though ion Pb^2+^ was not detected in EDX of ECHBS after biosorption, it is noteworthy that the first peak recorded in XRD (Fig. 13) was shifted after the biosorption. The first peak shifted to the lower 2θ after the biosorption, which indicated the enlarged structure of the ECHBS due to Pb^2+^ ion.

### FTIR (Complexation of the Functional Group)

**Figure 14:**
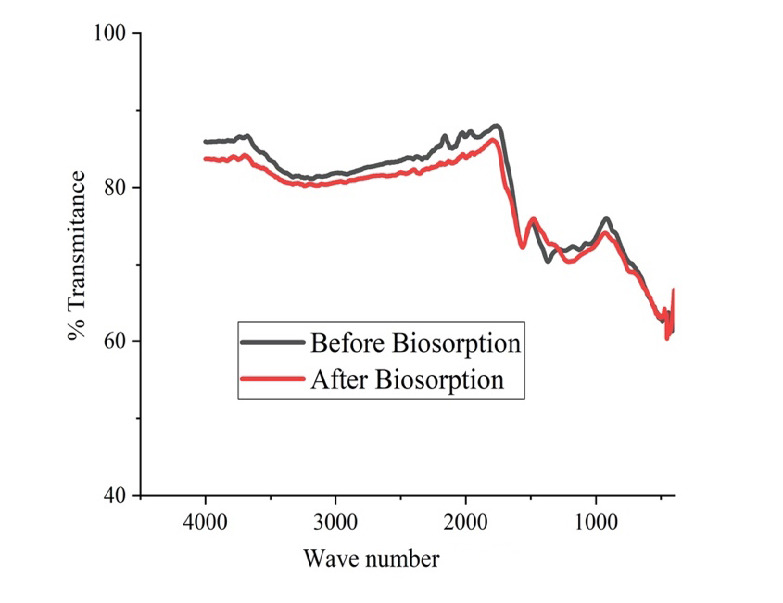
FTIR spectrums of ECHBS before and after adsorption of ion Pb^2+^.

Identifying surface functional groups of BS is necessary as it is the main chemical factor affecting adsorption of heavy metals by BS. The spectra of ECHBS before and after biosorption are presented in Fig. 14.

Based on Fig. 14, there was a slight change in the functional groups of ECHBS before and after biosorption. The bands around 3,500–3,200 cm^−1^, 1,700–1,600 cm^−1^, 1,500–1,400 cm^−1^ were detected from ECHBS before biosorption. These belong to -OH, C=O stretch and H-CH bend. Whereas the Pb^2+^ spectrum after biosorption showed that the width of the first two bands were changed, which belonged to the functional groups of -OH and C=O stretch. In addition, the position of the peak at 1,500–1,400 cm^−1^ was slightly shifted to 1,300–1,200 cm^−1^. According to this analysis, biosorption of Pb^2+^ by ECHBS is related to the functional groups of -OH, C=O, and H-CH. The obtained spectrums confirm that the surface of ECHBS was occupied by the heavy metal ion, and this caused the decrease in transmittance of the specified bands after biosorption.

### Comparison with Other Biosorbent on Pb^2+^

The adsorption capacity of biochar from ECH for the removal of Pb^2+^ ion in this study was compared with other adsorbate from CH as reported in the literature. The values of adsorption capacities are presented in Table 6. It can be seen that the experimental data of the present study had lower adsorption capacity compared to the referred adsorbates from CH.

## CONCLUSION

The ECH exhibited a great potential as a low-cost BS for removal Pb^2+^ ion from aqueous solution. According to the removal efficiency (%RE), ECH without chemical activation was chosen as the best method for using ECH as BS. Heating temperature, heating time and heating rate during the carbonisation process were considered as the critical parameters affecting the biosorption properties of the biochar from ECH (ECHBS). The study showed that the optimum heating time for ECHBS was 500°C. The highest adsorption capacity of ECHBS was achieved at heating time of 60 min and heating rate of 15°C/min. At the optimum conditions, the highest RE was 99%, while the biosorption capacity was 3.3 mg/g. The SEM EDX, FTIR, XRD and BET analysis on ECHBS before and after biosorption of Pb^2+^ ion showed that the mechanisms of ECHBS are mainly through ion exchange reaction of Pb^2+^ ion with K^+^ and Ca^2+^ metal ions. The adsorption equilibrium data followed the Langmuir isotherm, while the pseudo-second-order was determined as the better fit for kinetics model compared to the pseudo-first-order. The result showed that ECHBS can be a potential low-cost BS for removal ion Pb^2+^ from aqueous solution.

## Figures and Tables

**Table 1:** Type of separation factor (R_L_) of Langmuir isotherm.

R_L_ value	Type of isotherm
R_L_ > 1	Unfavourable
R_L_ = 1	Linear
0 < R_L_ < 1	Favourable
R_L_ = 0	Irreversible

**Table 2:** Porosity from BET analysis of ECH with different activating agent.

Activated carbon	Activating agent	Surface area (m^2^/g)	Pore size (nm)
This study ECH	H_3_PO_4_	943.33	3.33
This study ECH	No activating agent	7.87	41.19
This study ECH	ZnCl_2_	1.50	52.08
This study ECH	KOH	2.21	27.29
Ayalew and Aragaw (2020) CH	H_3_PO_4_	28.54	1.10
Oliveira *et al*. (2009) CH	ZnCl_2_	1,522.00	0.90

**Table 3:** Langmuir and Freundlich isotherm parameters.

Models	Langmuir			Freundlich		
	Qm (mg/g)	Kl (L/mg)	R^2^	Kf (mg/g)	*n*	R^2^
Isotherm constants	1.9	2.2	0.9521	27.183	8	0.5821

**Table 4:** Pseudo-first-order and pseudo-second-order adsorption of Pb^2+^ over ECHBS.

Adsorbent type	Initial Pb^2+^ concentration (mg/L)	Pseudo-first-order			Pseudo-second-order		
		Qe (mg/g)	K_I_ (min^−1^)	R^2^	Qe (mg/g)	K_2_ (g/mg min)	R^2^
ECH	20	2.72	0.03	0.8904	1.28	2.34	0.9376

**Table 5:** EDX elemental analysis of ECHBS before and after adsorption of ion Pb^2+^.

Element (Wt%)	Biochar (ECHBS)	ECHBS-Pb^2+^
C	76.96	78.52
O	19.11	20.59
S	0.28	-
K	2.96	0.89
Ca	0.68	-

**Table 6:** Comparison of Pb^2+^ adsorption capacity with other reported adsorbents from CH.

Adsorbent adsorption	Capacity (mg/g)	References
CH biomass waste	37.04	Alhogbi (2017)
Acid untreated CH	7.20	Alemayehu *et al*. (2014)
Untreated CH	50.80	Berhe *et al*. (2015)
Biochar from ECH	1.90	Present work
